# Supplementary material for: Early warning signals do not predict a warming-induced experimental epidemic
Source: PLOS Glob Public Health. 2025 Oct 8;5(10):e0005142. doi: 10.1371/journal.pgph.0005142 (PMC12507300; doi:10.1371/journal.pgph.0005142)
Supplement: S2 Fig — Raw data are shown in grey. Pre-processed time series are shown in blue (control populations) and red (warming populations). These data were pre-processed with a Gaussian kernel, with a bandwidth of three. A subset of 200 simulated time series is shown in each of the top panels; experimental time series are shown in the bottom panels. (PDF) [file pgph.0005142.s002.pdf]

S2 Fig: Empirical and simulated time series pre-processed with bandwidth of three.

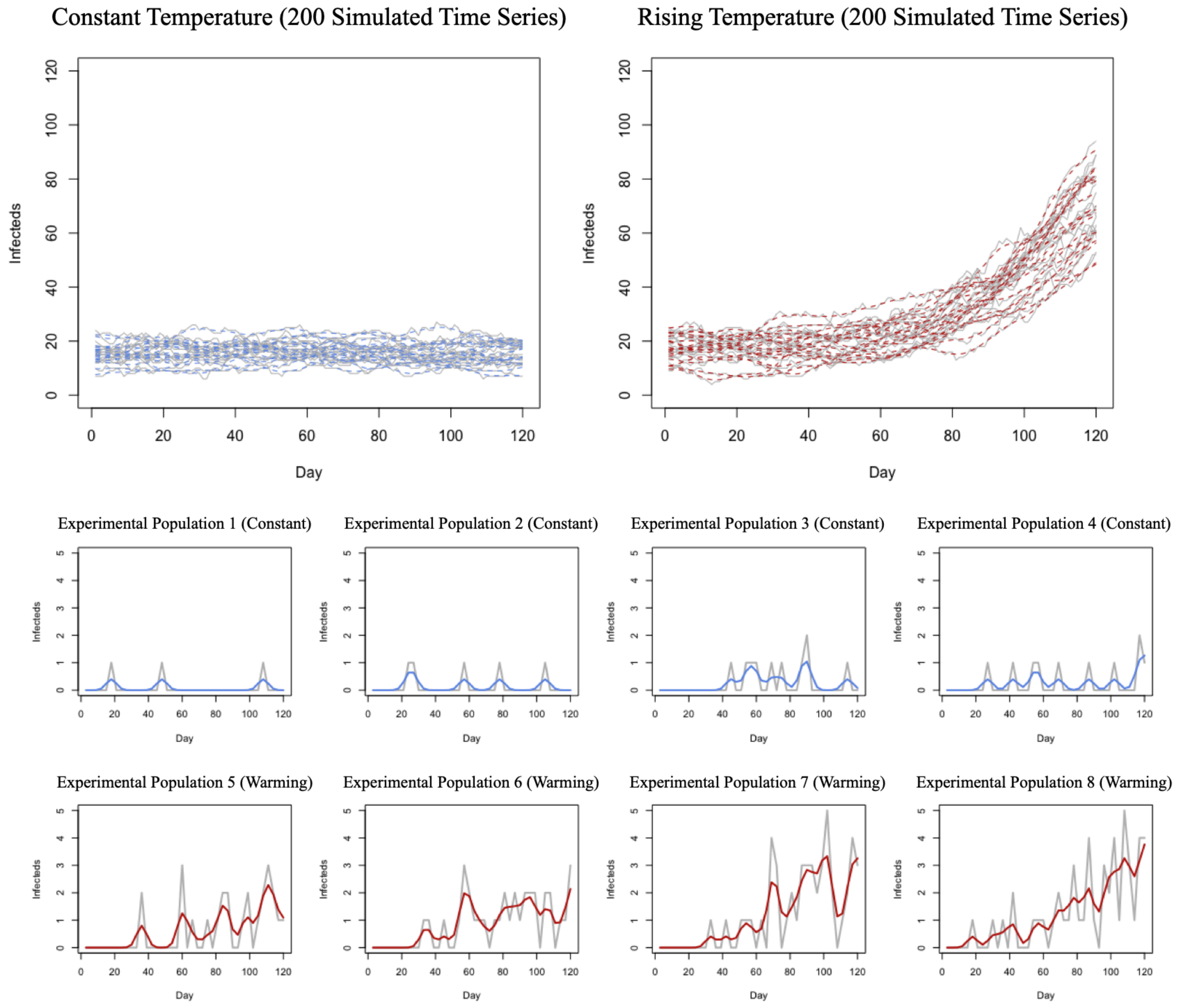

S2 Fig: Raw data are shown in grey. Pre-processed time series are shown in blue (control populations) and red (warming populations). These data were pre-processed with a Gaussian kernel, with a bandwidth of three. A subset of 200 simulated time series is shown in each of the top panels; experimental time series are shown in the bottom panels. Simulated time series show the total number of infecteds in a population, while experimental time series show the number of infecteds in a sample of twelve individuals.
