# Supplementary material for: Early warning signals do not predict a warming-induced experimental epidemic
Source: PLOS Glob Public Health. 2025 Oct 8;5(10):e0005142. doi: 10.1371/journal.pgph.0005142 (PMC12507300; doi:10.1371/journal.pgph.0005142)
Supplement: S6 Fig — In (A), (B), and (C), control populations are shown in blue, and warming populations are shown in pink. (A) Per the autoregressive model described in the main text (Eqn. (2.1)), trace plots showing sampled values of β1 (lag-1 autocorrelation of the residual time series) and σ (variance of the time series) across four chains, over time. (B) Posterior distributions of μ (mean of the time series). (C) Experimental times series overlaid atop model estimated time series that were used to generate a null distribution of trend coefficients. In (D), we show resulting null distributions of trend coefficients. (PDF) [file pgph.0005142.s006.pdf]

S6 Fig: Posterior visualizations of autoregressive model with Poisson process.

A

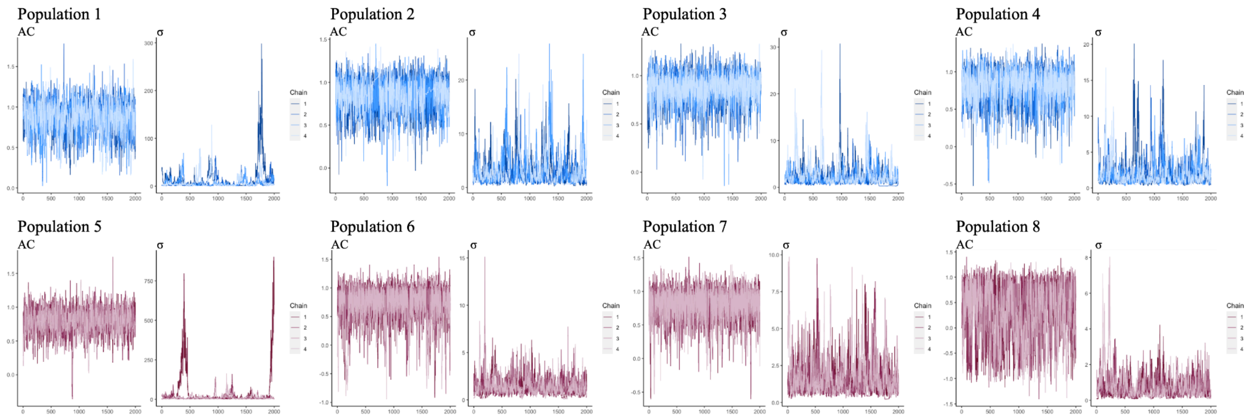

B

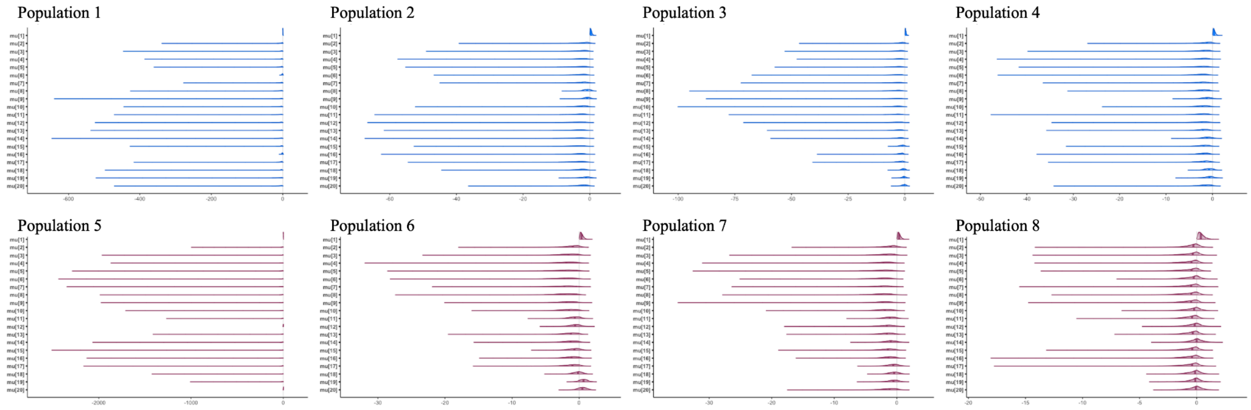

C

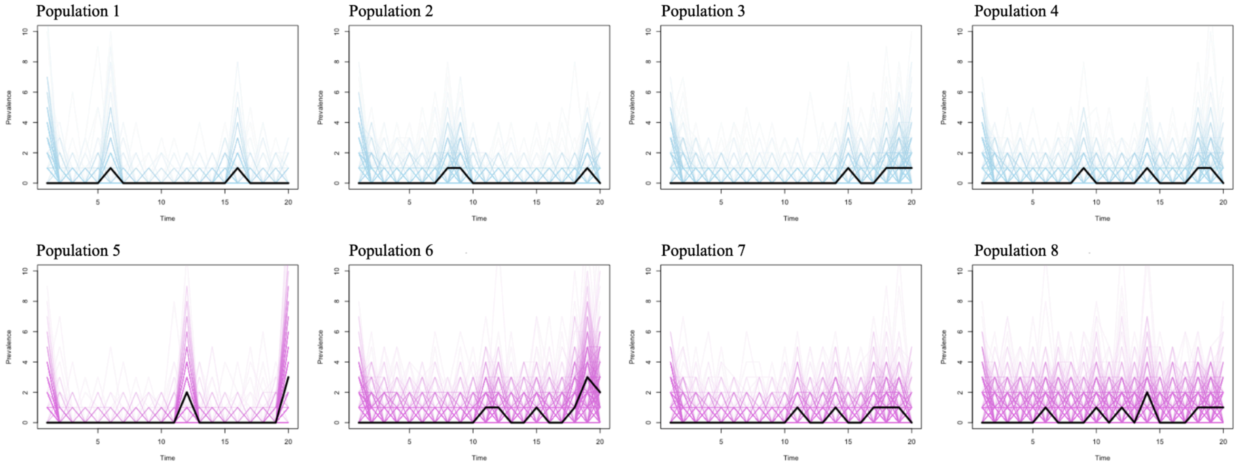

D

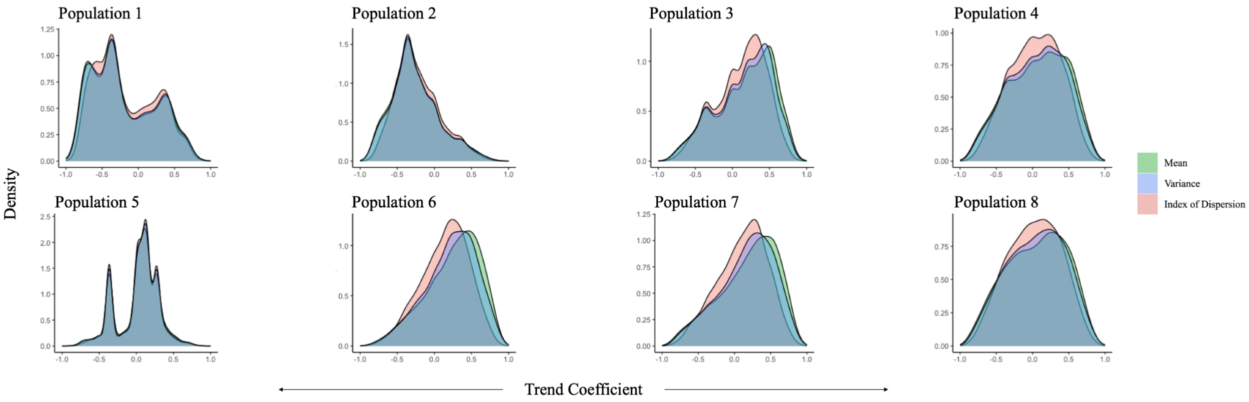

S6 Fig: In (A), (B), and (C), control populations are shown in blue, and warming populations are shown in pink. (A) Per the autoregressive model described in the main text (Eqn. (2.1)), trace plots showing sampled values of β_1 (lag-1 autocorrelation of the residual time series) and σ (variance of the time series) across four chains, over time. (B) Posterior distributions of μ (mean of the time series). (C) Experimental times series overlaid atop model estimated time series that were used to generate a null distribution of trend coefficients. In (D), we show resulting null distributions of trend coefficients.
