# Supplementary material for: Early warning signals do not predict a warming-induced experimental epidemic
Source: PLOS Glob Public Health. 2025 Oct 8;5(10):e0005142. doi: 10.1371/journal.pgph.0005142 (PMC12507300; doi:10.1371/journal.pgph.0005142)
Supplement: S7 Fig — Trend coefficients resulting from analysis of four experimental populations (1–4) maintained under constant conditions (blue), and four experimental populations (5–8) subjected to a warming treatment (pink). Statistical metrics were calculated within fifteen-day sliding windows. To evaluate statistical trends, we calculated Kendall’s rank correlation coefficient during the pre-critical interval (forty days (top panel), thirty days (bottom panel), and compared control (constant temperature, non-epidemic) and warming (warming treatment, epidemic emergence) coefficients across simulations and experimental populations by calculating the area under the curve (AUC) statistic. Values less than 0.5 indicate that a decrease in the indicator indicates emergence, while values greater than 0.5 indicate an increasing trend, with more extreme values indicating stronger trends. AUC statistics are shown on the vertical axis. EWS are shown on the horizontal axis. (PDF) [file pgph.0005142.s007.pdf]

S7 Fig: Trend coefficients resulting from analysis of experimental time series within different pre-critical intervals.

Days 20 to 60 (15 Day Sliding Window)

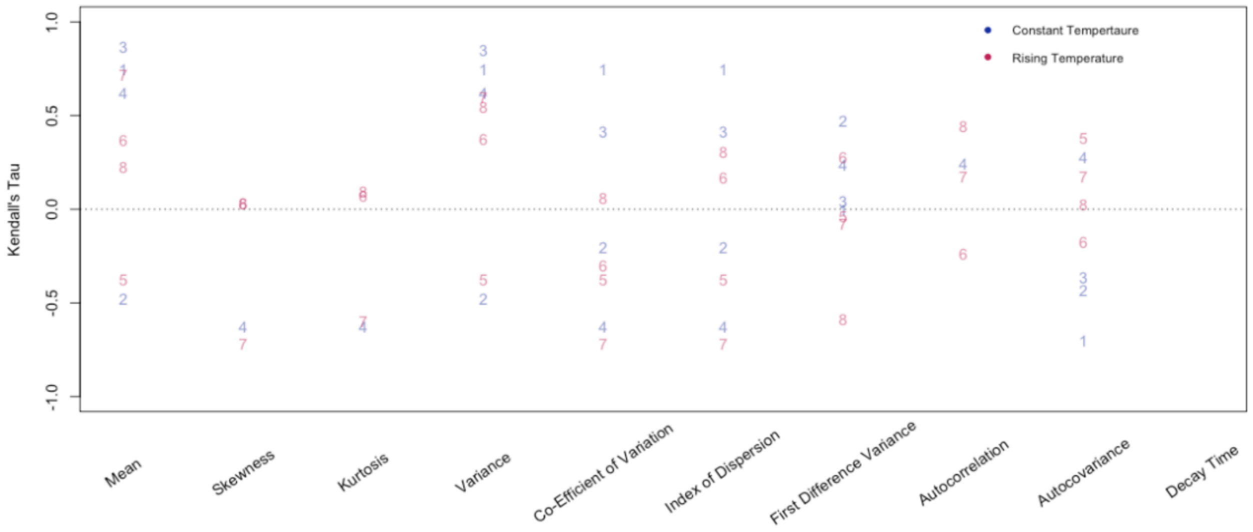

Days 30 to 60 (15 Day Sliding Window)

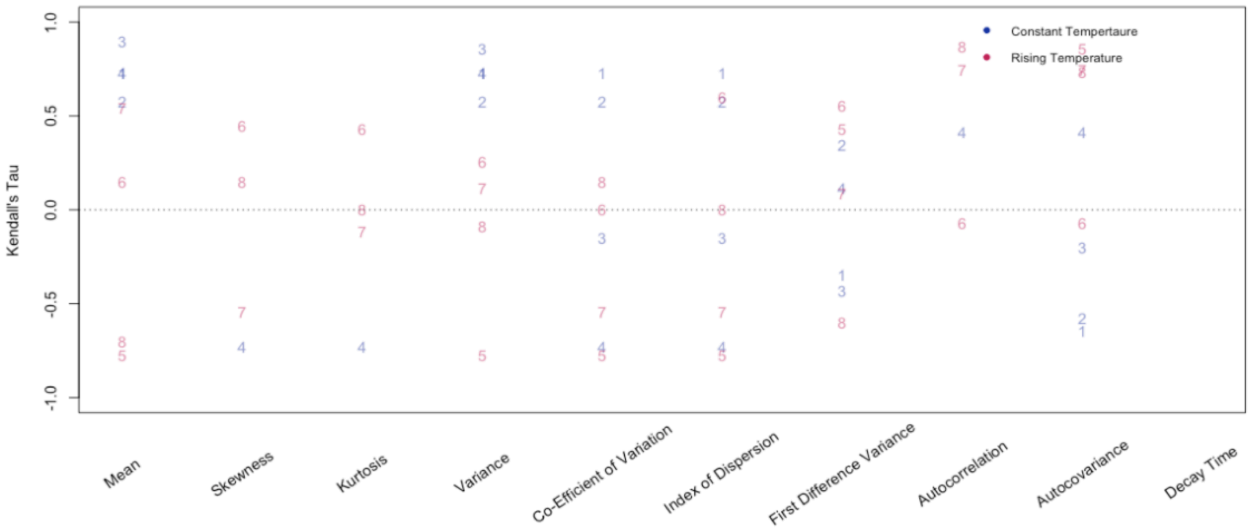

S7 Fig: Trend coefficients resulting from analysis of four experimental populations (1–4) maintained under constant conditions (blue), and four experimental populations (5–8) subjected to a warming treatment (pink). Statistical metrics were calculated within fifteen-day sliding windows. To evaluate statistical trends, we calculated Kendall's rank correlation coefficient during the pre-critical interval (forty days (top panel), thirty days (bottom panel), and compared control (constant temperature, non-epidemic) and warming (warming treatment, epidemic emergence) coefficients across simulations and experimental populations by calculating the area under the curve (AUC) statistic. Values less than 0.5 indicate that a decrease in the indicator indicates emergence, while values greater than 0.5 indicate an increasing trend, with more extreme values indicating stronger trends. AUC statistics are shown on the vertical axis. EWS are shown on the horizontal axis.
