# Supplementary material for: Early warning signals do not predict a warming-induced experimental epidemic
Source: PLOS Glob Public Health. 2025 Oct 8;5(10):e0005142. doi: 10.1371/journal.pgph.0005142 (PMC12507300; doi:10.1371/journal.pgph.0005142)
Supplement: S8 Fig — Density plots show the results of one thousand simulations under constant conditions (blue), and one thousand simulations under warming conditions (pink). Density is shown on the vertical axis and trend coefficients are shown on the horizontal axis. Vertical lines represent medians. Statistical metrics were calculated within fifteen-day sliding windows, during sixty-, forty-, and thirty-day pre-critical intervals. The first panel replicates the density plots shown in Fig 3, for reference. While different pre-critical intervals produced comparable overlaps between control and warming trend coefficients, we observed that smaller pre-critical intervals produced larger differences between median control and warming trend coefficients. During the first few days of the experiment, prevalence in control and warming populations was driven by immigration, rather than transmission. Later, and as the system warmed, we expected prevalence in warming populations to be driven by transmission, creating a disparity in prevalence between control and warming populations. As such, smaller pre-critical intervals, which excluded the first fifteen to thirty days of the experiment, produced larger differences between median control and warming trend coefficients. (PDF) [file pgph.0005142.s008.pdf]

S8 Fig: Density plots showing trend coefficient distributions within different pre-critical intervals.

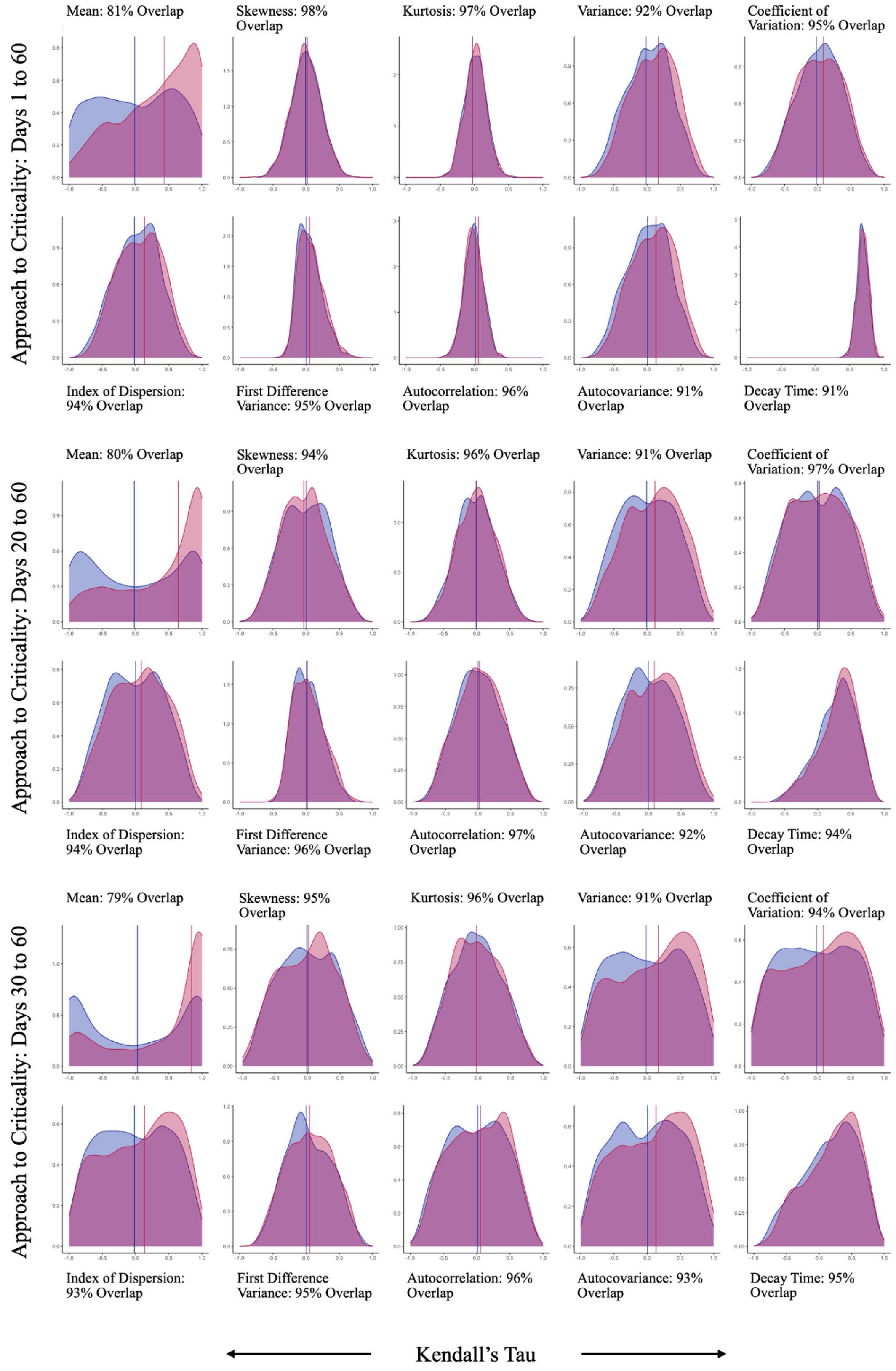

S8 Fig: Density plots show the results of one thousand simulations under constant conditions (blue), and one thousand simulations under warming conditions (pink). Density is shown on the vertical axis and trend coefficients are shown on the horizontal axis. Vertical lines represent medians. Statistical metrics were calculated within fifteen-day sliding windows, during sixty-, forty-, and thirty-day pre-critical intervals. The first panel replicates the density plots shown in Fig. 3, for reference. While different pre-critical intervals produced comparable overlaps between control and warming trend coefficients, we observed that smaller pre-critical intervals produced larger differences between median control and warming trend coefficients. During the first few days of the experiment, prevalence in control and warming populations was driven by immigration, rather than transmission. Later, and as the system warmed, we expected prevalence in warming populations to be driven by transmission, creating a disparity in prevalence between control and warming populations. As such, smaller pre-critical intervals, which excluded the first fifteen to thirty days of the experiment, produced larger differences between median control and warming trend coefficients.
