# Supplementary material for: Early warning signals do not predict a warming-induced experimental epidemic
Source: PLOS Glob Public Health. 2025 Oct 8;5(10):e0005142. doi: 10.1371/journal.pgph.0005142 (PMC12507300; doi:10.1371/journal.pgph.0005142)
Supplement: S9 Fig — To evaluate statistical trends, we calculated Kendall’s rank correlation coefficient during the pre-critical interval, and compared control (constant temperature/non-epidemic) and warming (warming treatment/epidemic emergence) coefficients across simulations and experimental populations by calculating the area under the curve (AUC) statistic. Values less than 0.5 suggest that a decrease in the statistical metric indicates emergence, while values greater than 0.5 suggest that an increase in the statistical metric indicates emergence, with more extreme values indicating stronger trends. AUC statistics are shown on the vertical axis. EWS are shown on the horizontal axis. Analyses were performed within fifteen-day sliding windows, between days one and sixty. (PDF) [file pgph.0005142.s009.pdf]

**S9 Fig:** AUC statistics as calculated from simulated data after accounting for the effects of sampling.

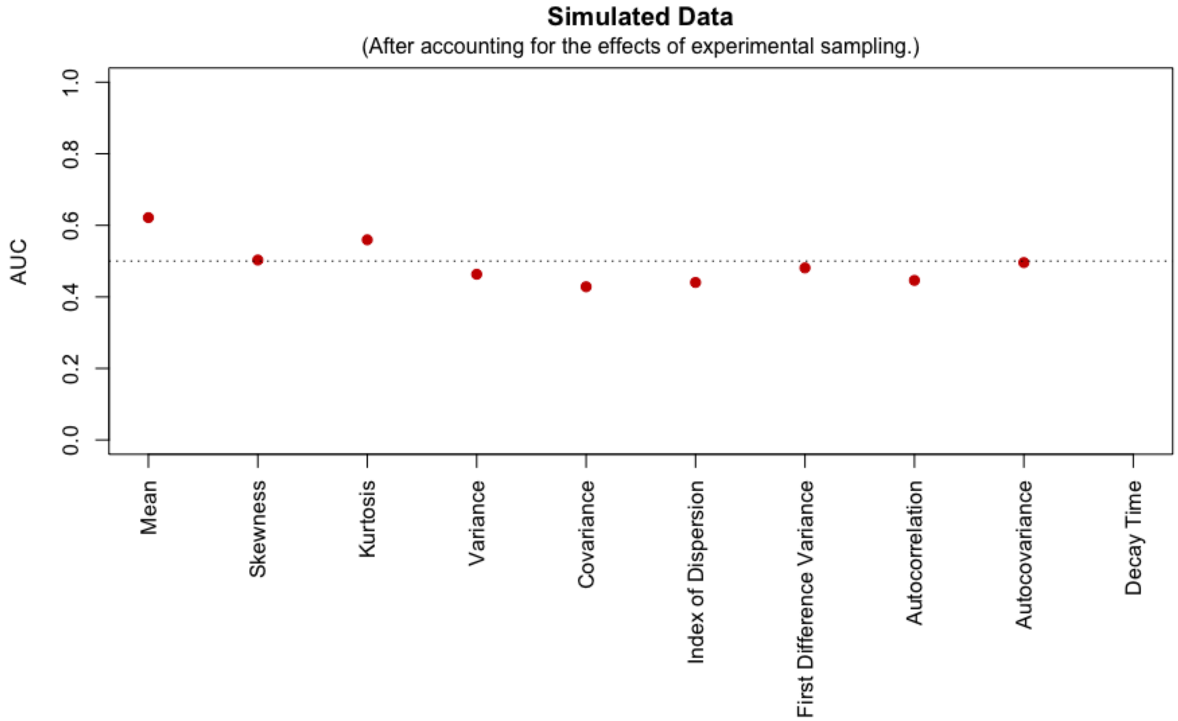


S9 Fig: To evaluate statistical trends, we calculated Kendall's rank correlation coefficient during the pre-critical interval, and compared control (constant temperature/non-epidemic) and warming (warming treatment/epidemic emergence) coefficients across simulations and experimental populations by calculating the area under the curve (AUC) statistic. Values less than 0.5 suggest that a decrease in the statistical metric indicates emergence, while values greater than 0.5 suggest that an increase in the statistical metric indicates emergence, with more extreme values indicating stronger trends. AUC statistics are shown on the vertical axis. EWS are shown on the horizontal axis. Analyses were performed within fifteen-day sliding windows, between days one and sixty.
