# Supplementary material for: Early warning signals do not predict a warming-induced experimental epidemic
Source: PLOS Glob Public Health. 2025 Oct 8;5(10):e0005142. doi: 10.1371/journal.pgph.0005142 (PMC12507300; doi:10.1371/journal.pgph.0005142)
Supplement: S1 Table — Blue text denotes control populations, while red text denotes warming populations. While median tau values are also available in the main text (Table 5), here, we additionally report AUC statistics to directly compare control and warming populations. Each statistical metric was calculated within five-, fifteen-, and thirty-day sliding windows, during sixty-, forty-, and thirty-day pre-critical intervals. To evaluate trends in these metrics, we calculated a median trend coefficient during the pre-critical interval over one thousand control and one thousand warming time series. Negative values indicate a decreasing trend prior to local bifurcation, while positive values indicate an increasing trend prior to local bifurcation. We compared control (constant temperature/non-epidemic) and warming (warming treatment/epidemic emergence) coefficients across simulations and experimental populations by calculating the area under the curve (AUC) statistic. Values less than 0.5 suggest that a decrease in the statistical metric indicates emergence, while values greater than 0.5 suggest that an increase in the statistical metric indicates emergence, with more extreme values indicating stronger trends. (PDF) [file pgph.0005142.s010.pdf]

S1 Table: Median trend coefficients and AUC statistics as calculated from simulated time series[a]. While median tau values are also available in the main text (Table 5), here, we additionally report AUC statistics to directly compare control and warming populations.

	Sliding Window: 5 Days						Sliding Window: 15 Days						Sliding Window: 30 Days	
	Days 1 to 60		Days 20 to 60		Days 30 to 60		Days 1 to 60		Days 20 to 60		Days 30 to 60		Days 1 to 60	
	Median Tau	AUC	Median Tau	AUC	Median Tau	AUC	Median Tau	AUC	Median Tau	AUC	Median Tau	AUC	Median Tau	AUC
Mean	0.008	0.670	-0.027	0.686	0.002	0.673	-0.011	0.651	-0.019	0.666	0.028	0.667	0.023	0.656
	0.329		0.438		0.474		0.410		0.643		0.843		0.693	
Skewness	0.004	0.527	0.005	0.503	0.000	0.502	-0.018	0.496	0.003	0.483	-0.008	0.492	-0.025	0.490
	0.010		0.002		0.000		-0.015		-0.034		0.017		-0.046	
Kurtosis	0.001	0.521	0.010	0.501	-0.003	0.537	0.002	0.500	-0.008	0.511	-0.025	0.498	-0.009	0.513
	0.010		0.007		0.013		-0.002		0.002		-0.025		0.006	
Variance	-0.006	0.632	-0.004	0.597	-0.010	0.567	0.013	0.578	-0.006	0.564	-0.017	0.560	0.016	0.558
	0.084		0.075		0.076		0.133		0.118		0.167		0.176	
Coefficient of Variation	-0.004	0.492	-0.008	0.488	-0.003	0.483	0.013	0.504	0.000	0.514	-0.017	0.525	0.016	0.518
	0.003		-0.017		-0.013		0.015		0.028		0.084		0.092	
Index of Dispersion	0.003	0.553	-0.003	0.536	-0.008	0.518	0.027	0.541	0.002	0.539	-0.017	0.543	0.025	0.539
	0.035		0.024		0.025		0.087		0.085		0.133		0.145	
First Difference Variance	0.005	0.525	0.005	0.507	0.002	0.511	0.007	0.525	0.007	0.517	0.000	0.516	0.058	0.520
	0.010		0.005		0.006		0.017		0.019		0.050		0.089	
Autocorrelation	0.014	0.509	0.003	0.516	0.012	0.503	0.012	0.506	-0.002	0.513	0.013	0.523	0.039	0.500
	0.023		0.013		-0.006		0.017		0.022		0.059		0.030	
Autocovariance	-0.001	0.537	0.005	0.531	0.000	0.518	0.018	0.562	-0.006	0.549	0.004	0.549	0.023	0.549
	0.019		0.019		0.013		0.098		0.088		0.133		0.177	
Decay Time	NA	NA	NA	NA	NA	NA	0.610	0.532	0.323	0.528	0.217	0.528	0.784	0.492
	NA		NA		NA		0.620		0.354		0.267		0.779	

[a]Blue text denotes control populations, while red text denotes warming populations.

Each statistical metric was calculated within five-, fifteen-, and thirty-day sliding windows, during sixty-, forty-, and thirty-day pre-critical intervals. To evaluate trends in these metrics, we calculated a median trend coefficient during the pre-critical interval over one thousand control and one thousand warming time series. Negative values indicate a decreasing trend prior to local bifurcation, while positive values indicate an increasing trend prior to local bifurcation. We compared control (constant temperature/non-epidemic) and warming (warming treatment/epidemic emergence) coefficients across simulations and experimental populations by calculating the area under the curve (AUC) statistic. Values less than 0.5 suggest that a decrease in the statistical metric indicates emergence, while values greater than 0.5 suggest that an increase in the statistical metric indicates emergence, with more extreme values indicating stronger trends.
