# Supplementary material for: Early warning signals do not predict a warming-induced experimental epidemic
Source: PLOS Glob Public Health. 2025 Oct 8;5(10):e0005142. doi: 10.1371/journal.pgph.0005142 (PMC12507300; doi:10.1371/journal.pgph.0005142)
Supplement: S2 Table — Blue text denotes control populations, while red text denotes warming populations. To evaluate trends in these metrics, we calculated a median trend coefficient during the pre-critical interval over one thousand control and one thousand warming time series. Negative values indicate a decreasing trend prior to local bifurcation, while positive values indicate an increasing trend prior to local bifurcation. We compared control (constant temperature, non-epidemic) and warming (warming treatment, epidemic emergence) coefficients across simulations and experimental populations by calculating the area under the curve (AUC) statistic. Values less than 0.5 suggest that a decrease in the statistical metric indicates emergence, while values greater than 0.5 suggest that an increase in the statistical metric indicates emergence, with more extreme values indicating stronger trends. (PDF) [file pgph.0005142.s011.pdf]

S2 Table: Median trend coefficients and AUC statistics as calculated from simulated time series after accounting for effects of experimental sampling during the sixty-day pre-critical interval[a].

	Sliding Window: 15 Days	
	Median Tau	AUC
Mean	0.012	0.622
	0.386	
Skewness	0.021	0.503
	-0.031	
Kurtosis	0.017	0.560
	0.092	
Variance	0.000	0.463
	-0.058	
Coefficient of Variation	0.000	0.428
	-0.131	
Index of Dispersion	0.000	0.440
	-0.096	
First Difference Variance	0.011	0.481
	0.000	
Autocorrelation	0.015	0.446
	-0.058	
Autocovariance	0.013	0.496
	0.000	
Decay Time	NA	NA
	NA	

[a]Blue text denotes control populations, while red text denotes warming populations.

To evaluate trends in these metrics, we calculated a median trend coefficient during the pre-critical interval over one thousand control and one thousand warming time series. Negative values indicate a decreasing trend prior to local bifurcation, while positive values indicate an increasing trend prior to local bifurcation. We compared control (constant temperature, non-epidemic) and warming (warming treatment, epidemic emergence) coefficients across simulations and experimental populations by calculating the area under the curve (AUC) statistic. Values less than 0.5 suggest that a decrease in the statistical metric indicates emergence, while values greater than 0.5 suggest that an increase in the statistical metric indicates emergence, with more extreme values indicating stronger trends
