# Supplementary material for: Early warning signals do not predict a warming-induced experimental epidemic
Source: PLOS Glob Public Health. 2025 Oct 8;5(10):e0005142. doi: 10.1371/journal.pgph.0005142 (PMC12507300; doi:10.1371/journal.pgph.0005142)
Supplement: S1 Text — (PDF) [file pgph.0005142.s012.pdf]

Description of data pre-processing

As described in the main text, we used Gaussian detrending to pre-process the experimental and simulated time series. Gaussian detrending, sometimes referred to as Gaussian filtering or Gaussian smoothing, is a moving average technique that involves using a Gaussian kernel (Eqn. 1) to calculate a weighted mean over a selected bandwidth to remove noise and/or non-focal trends (e.g. seasonal trends). In more detail, at every data point x, one calculates the kernel function (Eqn. 1), and estimates x as the weighted mean of a specified number of data points (the bandwidth) on either side of x (where the kernel function determines weights). Broadly, kernels define the shape of the function used to take the average of neighboring points, and as such, Gaussian kernels lend a heavier weight to nearby data points. The bandwidth of the kernel defines the number of data points included in the weighted average. As such, narrower kernels risk maintaining unwanted trends, while wider kernels risk removing the signal.

$$(1)\ k(x,\ x_i) = exp(-\frac{||x-x_i||^2}{2b^2})$$

Where $i = 1, ..., M$, and represent the distance between the focal point, x, and some other point within the specified bandwidth, b[1].

We detrended the simulation and experimental time series using bandwidths of two (Fig. S1), three (Fig. S2), and four (Fig. S3). As shown in Fig. S4, when applied to the experimental data, detrending inflated the strength and/or changed the directionality of observed trends. When applied to the simulated data, detrending had very small effects on the magnitudes of observed statistical trends. Given that pre-processing is expected to decrease the strength of observed trends, that increases in focal metrics should precede a transcritical bifurcation, and that neither experimental nor simulated conditions should have been influenced by seasonality, we suspect that in this case, detrending the empirical and simulated data introduced artificial trends, and thus produced unreliable results.

[1] Xiahai Zhuang and Yipeng Hu, 'Statistical Deformation Model: Theory and Methods', in *Statistical Shape and Deformation Analysis* (Elsevier, 2017), 33–65, https://doi.org/10.1016/B978-0-12-810493-4.00003-1.
