# Supplementary material for: Early warning signals do not predict a warming-induced experimental epidemic
Source: PLOS Glob Public Health. 2025 Oct 8;5(10):e0005142. doi: 10.1371/journal.pgph.0005142 (PMC12507300; doi:10.1371/journal.pgph.0005142)
Supplement: Attachment — Submitted filename: ReviewerResponse_1.pdf [file pgph.0005142.s014.pdf]

Authors' Response to Reviewers

Re: PGPH-D-25-01180 ("Early warning signals do not predict a warming-induced experimental epidemic").

Author's note: Refer to the bolded text below for responses. The line numbers correspond with those in the revised manuscript with tracked changes.

Editor:

After careful consideration, we feel that it has merit but does not fully meet PLOS Global Public Health's publication criteria as it currently stands. Therefore, we invite you to submit a revised version of the manuscript that addresses the points raised during the review process.

I would suggest the authors to revise the manuscript by clarifying several aspects including rationale of the study design, model assumption, initial conditions and result interpretation for further consideration.

Reviewer 1:

The authors analyze experimental and simulated time series of disease spread within populations of the model disease system Daphnia magna–Ordospora colligata for early warning signals of climate-mediated epidemic emergence. They found that the detection of false positives may limit the reliability and utility of early warning signals as predictors of climate-mediated epidemic emergence. The topic is interesting. I have some comments.

Methods:

1. Line 130: Please replace "Fig. 1–2" with "Figs. 1–2" and review all similar instances.
   Thank you for identifying this error. We've corrected figure references throughout the text.
2. Line 155: Symbols in "temperature-dependent ( )" brackets is not visible.
   Again, thank you for identifying this error, we've included the parameter, *T*, within the brackets.
3. Line 179: Please provide parameter settings for stochastic simulations.
   We apologise for not being clear that the model was demographically stochastic, as implemented via the Gillespie algorithm, and so does not have parameter settings beyond those indicated in Table 1. In the revised manuscript (line 182), we try to improve clarity by writing: "To generate the simulated data, we conducted <u>demographically</u> stochastic simulations of the mechanistic model that matched the duration and environmental conditions of the experimental system." In the same paragraph, we've added a sentence (lines 189–192) to clarify our use of the Gillespie stochastic simulation algorithm: "Demographic stochasticity was introduced via the Gillespie algorithm using the GillespieSSA package in R version 4.2.2 (57). <u>The</u>

GillespieSSA package uses the model of interest (here, Eqns. (1.1)–(1.4)) to generate stochastic population trajectories. Neither the simulations nor the experiment included environmental stochasticity.”

4. Line 184: Is one thousand time series sufficient?
   Initially, we simulated a set of one thousand time series, and a set of ten thousand time series. We did not identify qualitative differences between these sets, and opted to move forward with the set of one thousand time series to decrease computing time.
5. Line 210: What is the purpose of using Gaussian kernel? Have you tried other kernels?
   On Page 2 of the Supplemental Material, we write: “... we used Gaussian detrending to pre-process the experimental and simulated time series. Gaussian detrending, sometimes referred to as Gaussian filtering or Gaussian smoothing, is a moving average technique that involves using a Gaussian kernel to calculate a weighted mean over a selected bandwidth to remove noise and/or non-focal trends (e.g. seasonal trends). In more detail, at every data point x, one calculates the kernel function, and estimates x as the weighted mean of a specified number of data points (the bandwidth) on either side of x (where the kernel function determines weights). Broadly, kernels define the shape of the function used to take the average of neighboring points, and as such, Gaussian kernels lend a heavier weight to nearby data points. The bandwidth of the kernel defines the number of data points included in the weighted average. As such, narrower kernels risk maintaining unwanted trends, while wider kernels risk removing the signal.”

   Applying a Gaussian filter to experimental, observed, or simulated time series is an established practice in the literature (Dakos et al., 2008, 2012; Harris et al., 2020; O’Regan & Drake, 2013; Pananos et al., 2017; Southall et al., 2021). We did not explore different detrending methods for two reasons. First, given Kirk et al.’s (2020) experimental design and the construction of the mechanistic model used to simulate time series, we have no reason to believe that long-term trends are present in our experimental or simulated time series. Second, and as stated in lines 214–218, applying a Gaussian filter inflated the strength and reversed the directionality of observed trends (suggesting that pre-processing introduced artificial patterns). As such, we used the raw experimental and empirical data to generate the results presented and discussed in the main text.
6. Line 221: How did you determine the sliding windows?
   We selected sliding windows based on the length of the pre-bifurcation time series (thirty days, forty days, or sixty days), and analysed the time series within differently-sized sliding windows to assess the sensitivity of outcomes to sliding window choice. We focused on a fifteen-day sliding window as temperatures were increased every fifteen days within warming populations. We’ve added a sentence (lines 228–231) to the methods section to state this explicitly: “We analysed the empirical data within fifteen-day sliding windows, as temperatures were increased every fifteen days in

warming populations, and analysed the simulated data within five-, fifteen-, and thirty-day sliding windows to assess the sensitivity of outcomes to sliding window choice (37)." As described in the Results section and shown in Fig. S4, our findings weren't qualitatively sensitive to sliding window choice.

7. Line 253: Have you tried other distributions, such as negative binomial?

   We had not tried other distributions, but like the idea of considering a negative binomial distribution. We've since used the first half (pre-bifurcation) of each time series to parameterize an autoregressive model (AR-1), which we combined with a negative binomial distribution to simulate one thousand non-negative, integer-valued surrogate time series per population. We found that Poisson- and negative binomial-generated surrogate time series and resulting *P*-values exhibited qualitative agreement.

   Given that generating surrogate data via a negative binomial distribution did not qualitatively alter outcomes or conclusions, we have opted to briefly describe this process in the revised manuscript (lines 285–287): "We also used a negative binomial distribution to simulate surrogate time series as described above. We found that both processes produced similar time series, and that resulting P-values led to the same conclusions."

8. Is it reasonable to additionally provide the confidence interval of AUC?

   In lines 309–316, we write: "... we compared control (constant temperature/non-epidemic) and warming (warming treatment/epidemic emergence) coefficients across simulations and experimental populations by calculating the area under the curve (AUC) statistic. The AUC statistic measures the overlap between null and warming trend coefficient distributions…". As such, we calculate a single AUC statistic per metric of interest by comparing null and warming trend coefficients. Given that this statistic is not an estimated value, we cannot provide a confidence interval. The trend coefficient distributions (Fig. 3) illustrate the spread of our findings.

Results:

9. Table 3: Authors should clarify the corresponding scenario of these results (e.g. the sliding window and pre-critical interval), in addition, you should explain what "Pop'n1"-"Pop'n8" stand for. According to Table 2, coefficient of variation = sqrt(variance) / mean, Index of dispersion = variance / mean, which did not coincide with the numbers in Table 3?

   Thank you for your comment. We have clarified the sliding window and pre-critical interval by specifying a fifteen-day sliding window, and adding one line to the table description: "Here, we show analyses of empirical time series performed within the sixty-day pre-critical interval. See analyses within the forty- and thirty-day pre-critical intervals in Fig. S6." We further clarify "Pop'n" by adding "... across four control populations ("Pop'n 1" to "Pop'n 4") and four warming populations ("Pop'n 5" to "Pop'n 8")..." to the table description.

   Table 3 does not report raw statistical metrics (like mean, variance, etc.), and as

such, cannot be used to calculate statistical metrics. Table 3 reports trend coefficients and AUC statistics. As described in the Methods, we used each experimental/simulated time series to calculate the statistical metrics of interest within sliding windows. We then calculated their ordinal association using Kendall's τ. The resulting trend coefficient is a measure of the association between the rank order of the observed statistical metric and its position in time. We can then compare control and warming trend coefficients using the area under the curve (AUC) statistic. The AUC statistic measures the overlap between null and warming trend coefficient distributions and can be interpreted as the probability that a given warming coefficient will be higher than a given null coefficient.

Discussion:

10. I would like to suggest authors to indicate the situations in which EWS is not a reliable indicator of climate-mediated epidemic emergence and the situations in which EWS may be a reliable indicator.

    Thank you for your comment. Throughout the discussion, we consider the impacts of data quality and transmission dynamics on the detectability of EWS. In response to your comment, and comments from Reviewer 2, we've revised the Discussion (lines 517–536) to speculate on the impacts of transmission mode and abiotic conditions on detectability.

Reviewer 2:

The authors attempt to demonstrate the inferior performance of early warning signals (EWS) as indicators of epidemic emergence, using a model disease system Daphnia magna–Ordospora colligata under control and warming conditions. However, I find that the current version of the manuscript requires several clarifications before it can be considered for publication.

Major Comments:

1. L116: The disease experimental model is outside my expertise, but I am curious about the rationale behind initiating the experiment by introducing only 3 randomly selected adult D. magna from infected stock populations. This design appears to introduce considerable stochasticity, potentially leading to unstable EWS estimates (as discussed around L423). Is there a specific reason for not introducing a larger number of infected individuals from the stock population to improve the stability and reliability of EWS estimates? Given that the population remains endemic below 12°C, increasing the initial infected count might not alter the disease state but could reduce stochastic noise.

   We thank the reviewer for raising this question. We have carefully considered introducing higher numbers of infected individuals, and have concluded that this would not be feasible. If we were to introduce a larger number of infecteds into our host population ($N \approx 170$), prevalence would be driven by those introductions, rather than by disease transmission. Additionally, disease persistence at low levels (as we expect to

see in control populations) is a partial consequence of these introductions, and thus must remain low in order for us to see disease spread in response to warming.

2. L179: I wonder whether the stochastic simulations of the mechanistic model are fully consistent with the experimental initial conditions. Specifically, Fig. S1 shows approximately 20 or fewer infected individuals, whereas the experimental setup began with only 3 infected D. magna at maximum. This discrepancy in initial infection numbers between the simulation and experiment could impact model validity and interpretation.

   In Figures S1 through S3, we are plotting the simulated data, where we have a total number of infecteds in the population, alongside the empirical data, where we're counting the number of infecteds in a sample of twelve individuals. We have clarified this in the figure descriptions by stating "Simulated time series show the total number of infecteds in a population, while experimental time series show the number of infecteds in a sample of twelve individuals." Please also note that we included Figures S1 through S3 to show the impact of detrending on simulated and empirical time series. Figure 2 is a better representation of the difference between model and experimental outcomes.

3. L191: One model assumption is that R0 = 1 at 12°C theoretically. However, in the experimental system, stochasticity may cause deviations and it is possible for R0 reaching 1 at comparable temperatures (e.g., 11.5°C). Would it be more appropriate to fit a compartmental model directly to the data to estimate the bifurcation time empirically for subsequent analyses?

   Thank you for this comment. We agree that the exact time and/or temperature at which epidemic spread begins is influenced by stochasticity, but we consider such variations to be expected. Kirk et al., 2018 (*PLOS Biol.*) and Kirk et al., 2019 (*Am. Nat.*) (References 6 and 13, respectively) fit this mechanistic model to experimental data across a range of temperatures to characterise the relationship between temperature and R_0 that motivates our project. Here, we are aiming to detect statistical signatures of an impending bifurcation from a sub-epidemic state to an epidemic state. We would expect to detect such signatures prior to bifurcation, regardless of variation in the exact timing of bifurcation. As demonstrated by the agreement between our experimental and simulated findings, we would not expect these variations to affect our findings or conclusions, making fitting unnecessary. In addition, fitting a compartmental model to directly estimate bifurcation time would be a major computational undertaking, and would be unlikely to produce an estimate of R_0 precise enough to reliably differentiate a critical threshold of 12ºC v.s., say, 11.5ºC.

4. L234: Please clarify that Kendall's tau should be the measure of the association between the observed value "of metrics" and its position in time. Given that the observed number of infected is heavily tied, are the derived metrics also subject to many ties? It would be important to clarify how ties are handled in the Kendall's tau calculations and to assess whether different tie-handling methods affect the results.

We used the 'stats' package in R version 4.2.2 to calculate Kendall's τ. When there are ties, Kendall's τ_b is calculated, as described by Kendall, 1945 (*Biometrika*). Kendall's τ_b is a variation of Kendall's τ that supports ties, as defined by the following equation:

$$\tau_b = \frac{n_c - n_d}{\sqrt{(n_0 - n_1)(n_0 - n_2)}}$$

Where:

n_c **= Number of concordant pairs;**

n_d **= Number of discordant pairs;**

$n_0 = \frac{n(n-1)}{2}$;

$n_1 = \sum_i t_i(t_i - 1)/2$;

$n_2 = \sum_j u_i(u_i - 1)/2$;

t_i **= Number of tied values in the ith group of ties for the empirical distribution of X;**

u_j **= Number of tied values in the jth group of ties for the empirical distribution of Y.**

We have added a brief description of how ties are dealt with, along with a reference to Kendall, 1945, to the text (lines 251–252).

5. L243: The empirical data consist of non-negative integers, but surrogate time series generated by autoregressive (AR) models, the alternative method by Hamed and Rao (L262), and bootstrapping (L273) may include negative values. Please clarify whether these surrogate time series were rounded to integers and how negative values were handled. Since these methods assume continuous or real-valued data, violating the integer constraint of empirical data could bias the null distribution.

It is true that when we generated surrogate time series via an autoregressive process, we encountered real-valued data. Initially, we rounded decimals to the nearest integer and excluded negative values, but later determined that resulting surrogate time series were no longer good representatives of the empirical time series, and thus biased the null distributions. We opted to include the real-valued data to maintain important properties of the empirical data (like the autocorrelation of the original empirical time series), and because three of four significance testing methods we used, (1) the AR-1 model with a Poisson process (which we included in to account for the fact that the AR-1 model, without a Poisson process, will produce real-valued data), (2) the modified Mann-Kendall trend test, and (3) generating surrogate time series via bootstrapping, produce non-negative integers. We have clarified this in the revised manuscript (lines 270–274): "While using an autoregressive model to generate surrogate time series produces real-valued data (rather than non-negative, integer-valued), we opted to include these data to maintain the autocorrelation of the empirical time series, and

because three of the four significance assessments we describe and use produce non-negative integers, offering important points of comparison.”

The modified Mann-Kendall trend test uses characteristics of the empirical data to produce a null distribution of trend coefficients, rather than surrogate time series. In the subsection “Assessing Significance of Empirical Detections of Early Warning Signals”, we write: “... we used an alternative method of calculating the trend coefficient described by Hamed and Rao (1998) to generate a null distribution. We generated each null distribution by drawing one thousand samples from a normal distribution with a mean of zero and variance given by Eqn 2.2, where n is the number of observations in the first half of each time series, and $\rho_s(i)$ is the autocorrelation of the ranks of the observed statistic (e.g. mean, variance, index of dispersion, etc.).” to explain how we produced these null distributions.

The surrogate time series generated by bootstrapping are re-drawn from the empirical data, and are thus comprised of non-negative integers. To clarify this point, we have specified that the bootstrapped time series are re-sampled from the empirical time series in the revised manuscript (lines 301–302).

6. L243: Furthermore, why was only an AR(1) model considered instead of higher-order autoregressive model? Besides, since temperature gradually increases in the warming population, the time series may be non-stationary. Incorporating drift terms or applying differencing in the AR(1) model might be more appropriate to capture underlying trends.

We assessed the significance of our empirical detections in four ways. In each case, we used the first half of each time series to generate null distributions of the trend coefficient, against which we compared the observed value of the trend coefficient. As such, the surrogate time series are meant to represent populations that retain some characteristics of a given empirical population, but within which an epidemic will not emerge.

We used the acf function in R version 4.2.2 to calculate the partial autocorrelation function of the first half of each empirical time series and identify the appropriate order of the autoregressive model. We determined that a first order autoregressive model would produce the best surrogate data. To clarify our methods, we’ve added a couple sentences to the revised manuscript (lines 258–259): “First, we calculated the partial autocorrelation function of the first half of each empirical time series and identified a first order autoregressive model as an appropriate starting point.” However, we appreciate your comment, and in response, have tried using the first half of each time series to parameterize a second order autoregressive model (AR-2), and generate surrogate time series. We found that the AR-1 and AR-2 generated *P*-values exhibited agreement. Given that generating surrogate data via a second order autoregressive process did not qualitatively alter outcomes or conclusions, we have opted to describe this process in the revised manuscript (lines 268–270): “In the interest of robustness, we also used a second order autoregressive model to simulate real-valued surrogate time

series, and found that resulting null distributions and P-values led to the same conclusions."

When fitting the autoregressive model to the first half of the empirical time series, we use a moving average technique to account for nonstationarity. To clarify this point, we have modified a sentence in the Methods section (lines 276–280).

7. L299: The statement that "both control and warming populations exhibited increases in mean prevalence and variance" may be misleading. Focusing on the control populations, only 1 out of 4 control populations showed such increases with statistical significance (p-value). This should be clarified to avoid overgeneralization.
   We agree with Reviewer 2's comment, and have amended the sentence (lines 326–328) to read: "The empirical data revealed that during the approach to criticality (days one to sixty), one control population, and between one and two warming populations exhibited increases in mean prevalence and variance (Table 3, Figs. 3–4)."
8. L365, L378: Despite the expected trends in Table 2, what might explain the lack of observed trends in most metrics in both simulations and experiments? A discussion of possible methodological reasons would be helpful.
   Thank you for your comment. We agree that potential methodological explanations warrant discussion. We have revised such a discussion in lines 462–478.

Minor Comments:

1. L223: Regarding the sliding window analysis, it is unclear why there was insufficient data to apply a 30-day sliding window. Does this imply non-overlapping windows? A sliding window with a 3-day shift, aligned with the experimental sampling frequency, could yield approximately 31 estimates over 120 days, or 22 if excluding overlap around day 60. Additionally, have you considered using a 6-day window (a multiple of 3) instead of 5 days?

   We were most interested in the fifteen-day sliding window as temperatures were increased every fifteen days within warming populations.

   <u>Re: "Does this imply non-overlapping windows?":</u> We used the SlidingWindow function from the 'evobir' package in R to calculate statistical metrics within sliding windows. The windows do overlap.

   <u>Re: "A sliding window with a 3-day shift, aligned with the experimental sampling frequency, could yield approximately 31 estimates over 120 days":</u> Given that we're interested in detecting early warning signals of an upcoming bifurcation, we only analysed the first half (sixty days) of the time series (as we expect rising temperatures to trigger a bifurcation around day sixty). Broadly, given some data, $x_1, x_2, x_3, x_4, ..., x_n$, (where n is the time point at which we expect the system to bifurcate) if y is the size of the sliding window, the final statistical metric calculated will include values between x_{n-y} and x_n. We have tried to clarify this point in lines 224–225.

   As such, when applying a thirty-day sliding window to the first half of the simulated time series, we end up with thirty observations. Since experimental samples were taken

every three days, when applying a thirty-day sliding window to the first half of the experimental time series, we end up with just ten observations. I.e., when applied to the experimental time series, the thirty-day window yields insufficient data (revised manuscript, lines 233–234).

Re: "Additionally, have you considered using a 6-day window (a multiple of 3) instead of 5 days?": We did not initially consider a six-day sliding window, as each sliding window would only contain two observations.

In the interest of robustness, we have since analysed the empirical data using six- and thirty-day sliding windows. While the six-day results show some resemblance to the fifteen-day results, the thirty-day results often exhibit an unexpected negative association between the statistical metric of interest and time, suggesting they may be unreliable. We have (1) added a description of this analysis to the Methods section (lines 235–238), and (2) included an accompanying figure in the supplemental material (Fig. S5).

2. L241: It would be more appropriate to refer to values as "observed" or "estimated" rather than "true", especially since "true" is used extensively throughout the manuscript outside simulation contexts where it may be misleading.

   We agree with your comment, and have replaced "true" with "observed" throughout the text.
3. L302: Figure 5, I suggest increasing the plot height for clearer visualization, as the distributions overlap extensively.

   Thank you for this suggestion! We're revised the plot to more clearly differentiate the different distributions.
4. L309: Figure 3, please specify in the caption which of the 4 proposed methods was used to generate the null distribution shown.

   The bottom panel of Figure 3, "Simulated Data", shows distributions of trend coefficients resulting from the analysis of simulated time series. The simulated time series were generated to perform the simulation experiment, and are thus not null distributions. We only assessed the significance of our empirical detections in the four ways you reference in your comment. We've amended the figure description (lines 338–351) to clarify. The comparison between our empirical detections and null distributions are shown in Figure 5.
5. L355: Table 3, please specify the criteria used to determine whether a trend was detected.

   In lines 249–251, we write: "Here, Kendall's τ is a measure of the association between the rank order of the observed value and its position in time. As such, negative values indicate a decreasing trend prior to local bifurcation, while positive values indicate an increasing trend prior to local bifurcation."

   In lines 309–316, we write: "The AUC statistic measures the overlap between null and warming trend coefficient distributions and can be interpreted as the probability that a given warming coefficient will be higher than a given null coefficient. As such,

values less than 0.5 suggest that a decrease in the statistical metric indicates emergence, while values greater than 0.5 suggest that an increase in the statistical metric indicates emergence, with more extreme values indicating stronger trends."

As such, in both cases, any value that deviates from zero (in the case of Kendall's τ) or 0.5 (in the case of AUC) indicates a trend, while values farther away from zero or 0.5 indicate stronger trends. We agree that the wording implies a threshold test, so have decided to (1) remove the bolded text, and (2) note in the figure description (lines 376–394) that three metrics (mean, variance, and the index of dispersion) exhibit particularly deviant AUC values, and were thus subjected to additional analysis via four different methods (as described in the subsection "Assessing Significance of Empirical Detections of Early Warning Signals").

6. L380: Figure S7, despite comparable overlap percentages, there is a divergence in density near 0 and 1 in the shorter period approaching bifurcation, especially in the null distribution for the mean. Could you provide an explanation for this pattern?

   Thank you for your comment. In response, we have provided an explanation in the figure caption, which reads: "While different pre-critical intervals produced comparable overlaps between control and warming trend coefficients, we observed that smaller pre-critical intervals produced larger differences between median control and warming trend coefficients. During the first few days of the experiment, prevalence in control and warming populations was driven by immigration, rather than transmission. Later, and as the system warmed, we expected prevalence in warming populations to be driven by transmission, creating a disparity in prevalence between control and warming populations. As such, smaller pre-critical intervals, which excluded the first fifteen to thirty days of the experiment, produced larger differences between median control and warming trend coefficients."
7. L424–429: Do you mean that the comparable number of samples identified in control and warming populations results in the inferior performance of EWS? Please clarify this point and include the explanation immediately after describing the data.

   Thank you for your comment. We interpreted your last major comment (8) to be related to this comment. We agree that our argument should have been clearer. In response, we have re-written the paragraph (lines 464–467) to that end.
8. Supplementary Page 1: Please separate figures and tables into distinct sections. Currently, tables are interspersed among figures which disrupts the flow and readability.

   We have re-ordered the Supplementary Material list figures before tables.
